# Skin-on-a-chip models: General overview and future perspectives

**DOI:** 10.1063/5.0046376

**Published:** 2021-07-08

**Authors:** I. Risueño, L. Valencia, J. L. Jorcano, D. Velasco

**Affiliations:** 1 Department of Bioengineering and Aerospace Engineering, Universidad Carlos III de Madrid (UC3M), 28911 Leganés (Madrid), Spain; 2 Instituto de Investigación Sanitaria Gregorio Marañón, 28007 Madrid, Spain

## Abstract

Over the last few years, several advances have been made toward the development and production of *in vitro* human skin models for the analysis and testing of cosmetic and pharmaceutical products. However, these skin models are cultured under static conditions that make them unable to accurately represent normal human physiology. Recent interest has focused on the generation of *in vitro* 3D vascularized skin models with dynamic perfusion and microfluidic devices known as skin-on-a-chip. These platforms have been widely described in the literature as good candidates for tissue modeling, as they enable a more physiological transport of nutrients and permit a high-throughput and less expensive evaluation of drug candidates in terms of toxicity, efficacy, and delivery. In this Perspective, recent advances in these novel platforms for the generation of human skin models under dynamic conditions for *in vitro* testing are reported. Advances in vascularized human skin equivalents (HSEs), transferred skin-on-a-chip (introduction of a skin biopsy or a HSE in the chip), and *in situ* skin-on-a-chip (generation of the skin model directly in the chip) are critically reviewed, and currently used methods for the introduction of skin cells in the microfluidic chips are discussed. An outlook on current applications and future directions in this field of research are also presented.

## I. SKIN STRUCTURE AND FUNCTIONS

The skin is the largest organ of the body, typically making up 15%–20% of total body weight, with an external surface area of 1.8 m^2^ in adults. The main functions of the skin are sensory, thermoregulatory, metabolic, and protective. As the physical barrier against the environment, it controls the passage of molecules and ions while providing protection against microorganisms, ultraviolet radiation, and toxic or mechanical agents.^1^ It is composed of many sensory receptors that continuously examine the environment. The skin also acquires a thermoregulatory role, keeping body temperature constant. As a metabolic function, for instance, skin cells synthesize vitamin D needed in many processes, such as calcium homeostasis, and decrease the risk of developing diseases such as osteoporosis, arthritis, fractures, muscle weakness, and cancers.^2^ Skin is a dynamic organ in a constant state of change, as cells of the outer layers are continuously lost and replaced by inner cells moving up to the surface.^1^

**FIG. 1.**
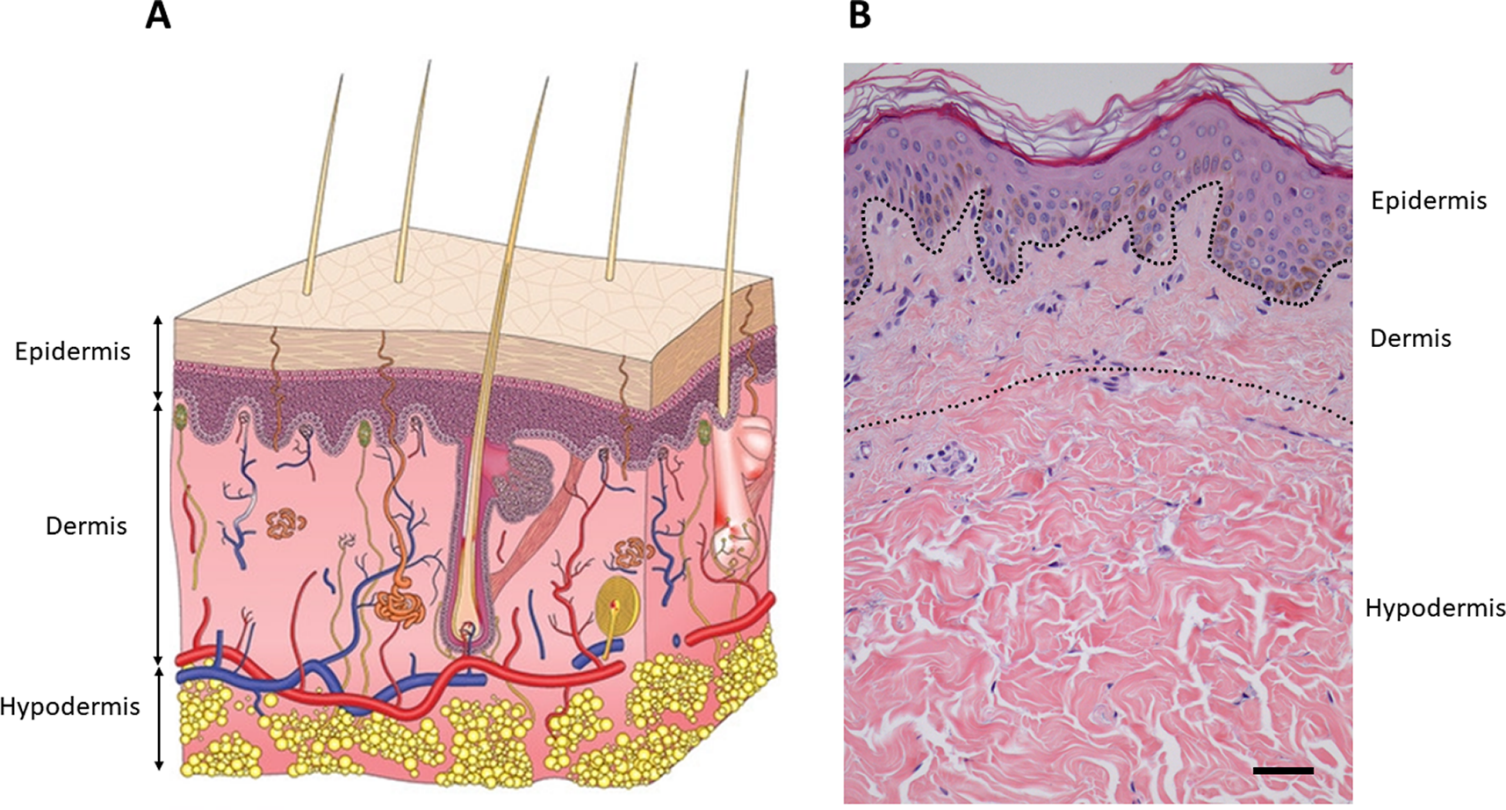
Skin structure. The outermost stratum is the epidermis, a stratified layer of keratinocytes; the dermis is found underneath, which consists of dense irregular connective tissues and cushions the body from stress and strain. Finally, the hypodermis is the innermost layer of the skin, mainly functioning as fat storage. (a) Schematic view. Modified image. Republished with permission from Sutterby *et al.*, Small **16**, 39 (2020). Copyright 2021 John Wiley and Sons, permission conveyed through Copyright Clearance Center, Inc.^5^ (b) Histological section. Modified image. Reprinted with permission from Mine *et al.,* PLoS One **3**, 12 (2018). Copyright 2021 Authors, licensed under a Creative Commons Attribution (CCBY) License 4.0.^6^

Human skin is composed of three structural layers: the epidermis, the dermis, and hypodermis (Fig. 1). The epidermis consists mainly of superimposed layers of keratinocytes that produce keratin as a protective protein. The middle layer, the dermis, is a connective tissue mainly composed of collagen embedding fibroblasts. The underlying subcutaneous tissue consists of fat cells (adipocytes) as the primary cellular component. The thickness of these layers depends on the part of the body where it is found. The thinnest epidermis is found at the eyelid and the thickest at the sole of the feet, varying from 0.1 to 1.5 mm, respectively.^3^ On the contrary, the thickest dermis is found at the back, being 30–40 times thicker than the epidermis.^4^

### A. Epidermis

The epidermis is the outermost stratified squamous keratinized layer of the skin composed of cells called keratinocytes,^2^ although it hosts other but less abundant cells such as melanocytes that produce the pigment melanin, Langerhans involved in an immune response, or Merkel cells entailed in tactile sensation.^7^ It is an important barrier between the organism and its environment, protecting it from physical, chemical, and microbial damage, and it also regulates the function and integrity of the underlying connective tissue.^8^

The epidermis is continuously renewed bringing about secondary structures such as sebaceous or sweat glands, nails, and hair follicles.^9^ At least 80% of the epidermal cells are keratinocytes, and its terminal differentiation process consists of cell migration from the basal layer to the surface, resulting in keratinization leading to loss of organelles and resulting in a mixture of filaments due to cell death.^3^ Epidermis is also divided into four layers due to keratinocyte differentiation and cornification: the basal layer (*stratum germinatum*), squamous layer (*stratum spinosum*), granular layer (*stratum granulosum*), and cornified layer (*stratum corneum*).

The basement membrane is the dermo-epidermal junction, mainly composed of type IV and VII collagens and laminin along with other proteins such as nidogen, perlecan, fibronectin, and proteoglicans that allow the correct exchange of substances.^10,11^ For a more detailed description, see Ref. 12. This layer provides support to the epidermis and enhances cell polarity and growth by releasing appropriate chemical signals.^3,13^

### B. Dermis

The dermis is relatively acellular compared to the epidermis. It is a complex system of fibrous connective tissues, mainly composed of type I and II collagens and elastic fibers (elastin). Collagen fibers provide mainly tensile strength, and the elastin main function is resilience and elasticity of the skin.^12^ Hyaluronic acid is also an important component of the dermal extracellular matrix (dECM) due to its function in skin hydration. Collagen, elastin, and hyaluronic acid composition, distribution, and arrangement are not uniform, and it is in constant remodeling and change.^3,10^ Aging has an important role in skin loss of elasticity associated with the disorganization and reduction of these main functional proteins.^6^

The most abundant cells are the fibroblasts, but it also hosts nerve and vascular networks, macrophages, mast cells, lymphocytes, adipocytes, Schwann cells, and stem cells. In addition, it also shelters blood vessels, sensory receptors, glands, hair follicles, and nerves.^9^ The dermis is the major component of the skin, provides elasticity, tensile strength, thermal regulation, and sensing capacity, and protects the body from mechanical damage.

### C. Hypodermis

Finally, the hypodermis consists of loose and well-vascularized connective tissues that join skin to subjacent organs and have larger nerves and blood vessels than those found in the dermis. It is mainly composed of adipocytes, fibroblasts, and macrophages.^2^ Its thickness varies depending on the individual gender and the region of the body ranging from 1.9 to 7.1 mm,^14^ being the thickest layer of the skin. It is considered as an endocrine organ and provides buoyancy, energy storage, and hormone conversion, playing an important role in thermal homeostasis.^10^

## II. ENGINEERED HUMAN SKIN EQUIVALENTS FOR *IN VITRO* TESTING

Nowadays, there is an increasing demand for the development and production of *in vitro* engineered skin models for either restoring its function after damage or cosmetic and pharmaceutical testing.^15^ Tissue-engineered skin substitutes market was valued at 958.8 million US dollars in 2014, and it is projected to reach 3873.5 million US dollars by 2023. Particularly, *in vitro* toxicology testing was valued at 14.2 billion US dollars in 2016. In the case of skin, many compounds cannot be directly tested on humans, so *in vivo* animal testing was a common technique to assess its efficacy and toxicity.^16^ However, since March 2013, animal use for testing of cosmetics has been banned in Europe as stated in the Directive 2003/15/EC of the European Parliament and of the European Council, 27 February 2013.^17^ In addition to the ethical implications, these models sometimes fail to predict human responses due to differences between human and animal physiologies, leading to costly, unsuccessful, and expensive clinical trials.^18^ At this stage, the new goal is the practice of the “3R” principle of Refinement, Reduction, and Replacement of animal tests whenever possible.^16,19^

**FIG. 2.**
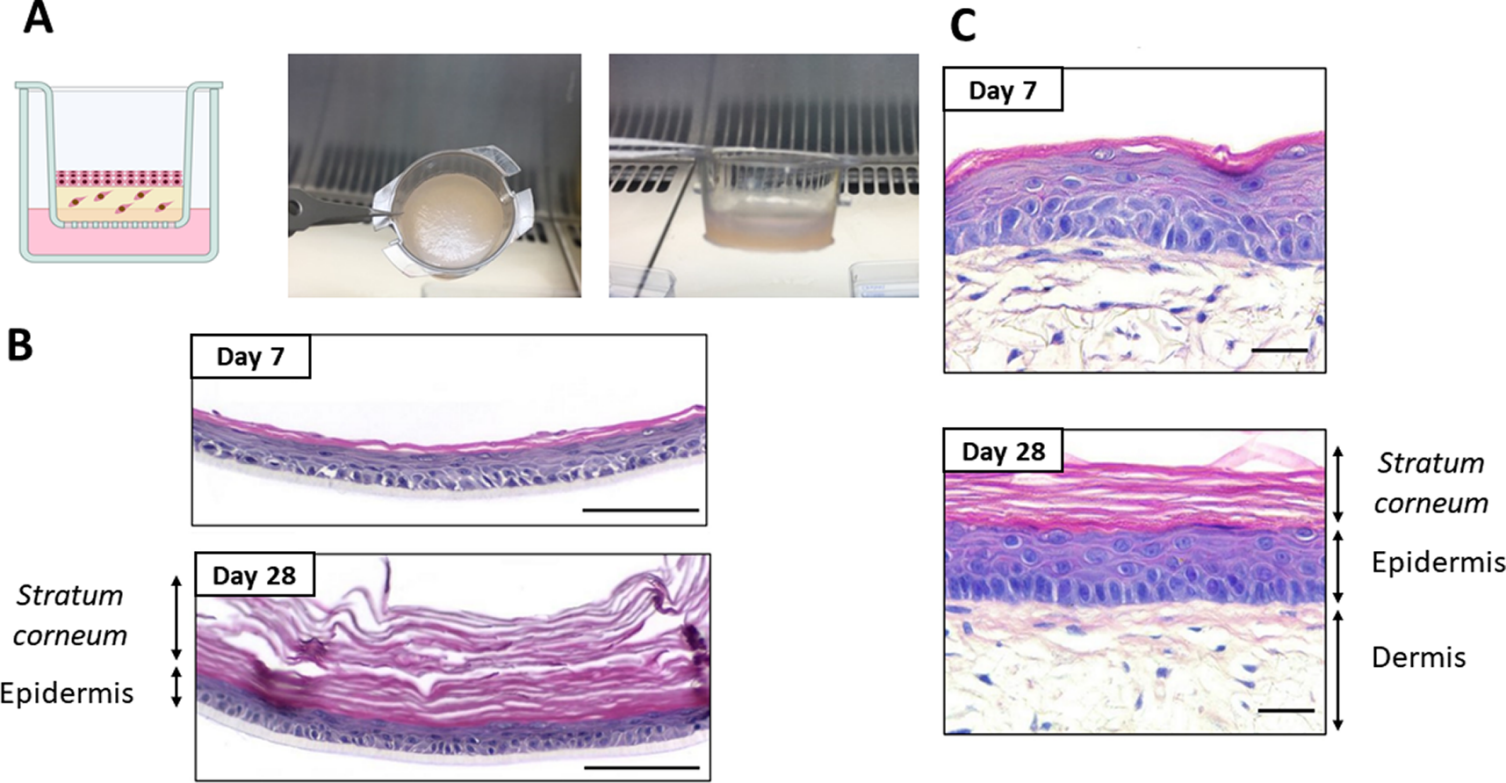
Engineered *in vitro* human skin equivalents. (a) Schematic and pictures of a dermo-epidermal human skin equivalent (HSE) cultured in a transwell insert. Images are kindly provided by Cristina Quílez from our group. (b) Epidermal skin equivalent at 7 and 28 days of culture at the air-liquid interface, scale bars: 100 *μ*m. (c) Dermo-epidermal skin equivalent at 7 and 28 days of culture at the air-liquid interface, scale bars: 30 *μ*m. (b) and (c) are modified images. Reprinted with permission from Roger *et al.*, J. Anat. **234**, 4 (2019). Copyright 2019 Authors, licensed under a Creative Commons Attribution (CCBY) License 4.0.^23^

Early *in vitro* skin models consisted of 2D cell monolayers, but continuous efforts lead to reconstructed human epidermal (RHE) models to better mimic skin layers and its biomolecular properties close to *in vivo* conditions,^20^ allowing researchers to perform reliably *in vitro* toxicological and cosmetic studies as alternatives to animal models.^21^ Among the available models, there are cellular skin substitutes including epidermal cell monolayers and dermo-epidermal bilayers (holding keratinocytes and fibroblasts) skin substitutes^15^ (Fig. 2). The development of artificial skin equivalents involves artificial and natural polymers for the generation of the scaffold such as alginate, collagen, chitosan, fibrin, hyaluronic acid, elastin poly (ethylene glycol), polycaprolactone, poly(vinyl alcohol), or polylactic acid. Collagen is the most abundant component of the skin, and its combination with other polymers provides better structural and mechanical properties.^22^

Three-dimensional organotypic skin models are commonly used in *in vitro* applications for cosmetic products testing or products evaluation in pharmaceutical industries, and they are already commercially available.^16,20,23^ Some of them reconstructed only the human epidermis like SkinethicTM (EpiSkin, L'Oréal Lyon France), the EST1000® skin model (CellSystems, Troisdorf, Germany), the OS-Rep (Open Source Reconstructed Epidermis) model (Henkel, Düsseldorf, Germany), the StratiCELL model (StratiCELL, Les Isnes, Belgium), the StrataTest® model (Stratatech, Madison, WI, USA), and, more recently, the LabCyte Epi-model (LabCyte, Gamagori, Japan). There are also other skin models that recapitulate both dermal and epidermal compartments such as the Vitrolife-Skin™ model (Kyoto, Japan), the Phenion® Full-Thickness skin model (Henkel, Düsseldorf, Germany), the EpiDerm-FT™ (Mattek, Ashland, USA), the CELLnTEC full thickness skin model (CELLnTEC, Berne, Switzerland), or the Biomimiq full thickness skin model (Biomimiq, Leiden, Netherland), among others.^9^

Since 2003, ECVAM (the European Center for the Validation of Alternative Methods) has focused on evaluating suitable reconstructed models to identify skin irritant and corrosive compounds in order to develop alternative tests to the approved *in vivo* ones.^24^ Some of these commercially available organotypic skins have been validated as irritation and corrosion testing methods by the OECD [OECD Test Guideline N°439 (*In Vitro* Skin Irritation: Reconstructed Human Epidermis Test Method) and OECD Test Guideline N°431 (*In Vitro* Skin Corrosion: Reconstructed Human Epidermis Test Method)].

Dermo-epidermal skin equivalents must fulfill certain conditions such as mechanical properties, well-differentiated epidermis with *stratum corneum*, and dermis and epidermis properly linked through the basal lamina. Some recent advances in skin engineering have produced skin equivalents that incorporate a wide variety of cell types and structures that better resemble the real structure of the organ.^15^ Incorporation of adipocytes, dermal papilla cells to induce hair follicles, endothelial cells to giving rise to vascularization, immune or Langerhans cells to reproduce immune response, chemokines to promote cell differentiation, or dorsal root ganglion neurons to recreate the peripheral skin nerve system are improvements to the current *in vitro* skin models to better mimic its response to irritation or toxicity studies.^20,25^ Nevertheless, the limited lifespan of reconstructed skin, due to rapid degradation and an excessive contraction exerted by the skin cells present in the culture, is still a persisting issue. Several strategies have been proposed to overcome this limitation such as modifying *in vitro* culture conditions or combining polymers to engineer scaffolds with better mechanical properties.^26–28^ For more details, we suggest the reader to see Refs. 9 and 29.

One of the main drawbacks of skin equivalents is still their lack of a vascular system in the sense of nutrients, oxygen supply, waste removal, or concentration gradient of the nutrients, trafficking of leukocytes and transdermal penetration of drugs to the bloodstream.^30^ A proper vascularization would allow an efficient nutrient and oxygen exchange, resulting in a longer survival of the tissue.^31^ The complexity of real human skin has not yet been accomplished regardless of the efforts to improve skin equivalents composition and structure. For *in vitro* skin model improvements, dynamic platforms could provide this transport mechanism for a more realistic drug delivery and toxicity studies.^32^ Here, we present an overview of current advances on dynamic skin models for *in vitro* testing, including artificially vascularized human skin equivalents (HSEs) and skin-on-a-chip devices. Their main characteristics are summarized in Table I.

**TABLE I.** Summary of the dynamic skin models reported in the literature. Abbreviations: dECM, decellularized extracellular matrix; HSE, human skin equivalent; HUVECs, human umbilical vein endothelial cells; iPSCs, induced pluripotent stem cells; PC, polycarbonate; PCL, polycaprolactone; PDMS, polydimethylsiloxane; PEG, polyethylene glycol; PET, polyethylene terephthalate; PMMA, poly(methyl methacrylate); PS, polystyrene; PVC, polyvinyl chloride.

	Device material	Flow	Cells	Membrane (material/pore size)	Dermal matrix	Type of platform
**Abaci *et al.* (2016)** ^59^	PDMS	Pumpless, gravity driven	Primary fibroblasts and keratinocytes	PC/5 *μ*m	Collagen	Transferred skin-on-a-chip
**Abaci *et al.* (2016)** ^38^	Commercial resin + transwell insert	Perfusion, simulated vasculature	Primary fibroblasts and keratinocytes + iPSCs for endothelial	PET/3 *μ*m	Collagen	Vascularized HSE
**Alexander *et al.* (2018)** ^69^	Commercial platform + transwell insert	Perfusion	L929 murine fibroblasts and EpiDerm™	Not stated/3 *μ*m	None	Transferred skin-on-a-chip
**Ataç *et al.* (2013)** ^71^	PDMS	Perfusion, on-chip micropump	EpiDermFT™ + *ex vivo* subcutaneous tissue	Not stated	EpiDermFT™ (commercial)	Transferred skin-on-a-chip^a^
**Jeon *et al.* (2020)** ^76^	PDMS	Pumpless, gravity driven	Primary fibroblasts and keratinocytes	Not stated/0.4 *μ*m	Collagen	*In situ* skin-on-a-chip
**Kim *et al.* (2019)** ^41^	PCL	Perfusion	Human fibroblasts and keratinocytes + HUVECs + human preadipocytes	None	dECM-based bioink + fibrinogen	Vascularized HSE
**Kim *et al.* (2019)** ^68^	PDMS	Static	Blood cells + human biopsy	Red blood cell filter	Biopsy	Transferred skin-on-a-chip
**Kim *et al.* (2020)** ^77^	PDMS	Pumpless, gravity driven	Primary fibroblasts and keratinocytes	Polyester/Not stated	Collagen	*In situ* skin-on-a-chip
**Lee *et al.* (2017)** ^73^	PDMS	Pumpless, gravity driven	Primary fibroblasts and keratinocytes + HUVECs	PC/Not stated	Collagen	*In situ* skin-on-a-chip
**Lim *et al.* (2018)** ^78^	PDMS and glass	Perfusion	Human fibroblasts and keratinocytes	Not stated	Collagen	*In situ* skin-on-a-chip
**Maschmeyer *et al.* (2015)** ^72^	PDMS	Perfusion, on-chip micropump	Human biopsy	Not stated/0.4 *μ*m	Biopsy	Transferred skin-on-a-chip^a^
**Mori *et al.* (2017)** ^39^	Not stated	Perfusion, simulated vasculature	Normal human fibroblasts and keratinocytes + HUVECs	None	Collagen	Vascularized HSE
**Mori *et al.* (2019)** ^40^	Flexible silicone rubber (PDMS and Ecoflex®)	Perfusion, simulated vasculature	Normal human fibroblasts and keratinocytes + HUVECs	None	Collagen	Vascularized HSE
**O'Neill *et al.* (2008)** ^79^	PDMS	Perfusion	Normal human keratinocytes	None	None	Microfluidic platform
**Ramadan *et al.* (2016)** ^82^	PMMA, PS, and PDMS	Perfusion (negative pressure)	Immortalized HaCaT keratinocytes and U937 for dendritic cells	PET/0.4 *μ*m	None	*In situ* skin-on-a-chip
**Risueño *et al.* (2021)** ^84^	Adhesive vinyl (PVC), PDMS, and glass	Perfusion	Primary human fibroblasts and immortalized HaCaT keratinocytes	PC/5 *μ*m	Fibrin	*In situ* skin-on-a-chip
**Sasaki *et al.* (2019)** ^81^	PDMS	Perfusion	Immortalized HaCaT keratinocytes	PET/1 *μ*m	None	*In situ* skin-on-a-chip
**Song *et al.* (2017)** ^74^	PDMS	Pumpless, gravity driven	Primary fibroblasts and keratinocytes	Not stated	Collagen (different sources)	*In situ* skin-on-a-chip
**Song *et al.* (2018)** ^75^	PDMS	Pumpless, gravity driven	Primary fibroblasts and keratinocytes	Not stated	Collagen	*In situ* skin-on-a-chip
**Sriram *et al.* (2018)** ^80^	PMMA	Perfusion	Primary fibroblasts and immortalized N/TERT keratinocytes	PC/1 *μ*m	Fibrin + PEG	*In situ* skin-on-a-chip
**Wagner *et al.* (2013)** ^70^	PDMS	Perfusion, on-chip micropump	Human biopsy	Not stated/0.4 *μ*m	Biopsy	Transferred skin-on-a-chip^a^
**Wufuer *et al.* (2016)** ^83^	PDMS	Pumpless, gravity driven	Immortalized HS27 fibroblasts and HaCaT keratinocytes + HUVECs	PET(×2)/0.4 *μ*m	None	*In situ* skin-on-a-chip

^a^ Multiorgan-on-a-chip. Data shown in the table correspond to the skin section of the device.

## III. VASCULARIZED HUMAN SKIN EQUIVALENTS

Regarding all the limitations present in the traditional HSEs, several research groups have worked in the development of different platforms integrating skin equivalents with a perfusable vasculature, allowing dynamic and more sophisticated systems. The addition of this vascularization has opened new research fields beyond the most common applications of static cultured constructs; these new models could be used for angiogenesis studies,^33^ evaluation of angiostatic drugs,^34^ or cancer research.^35^

Some approaches use stem or endothelial cells seeded inside collagen and fibrin hydrogels, which are later exposed to vascular endothelial growth factor (VEGF) to induce vascularization.^36,37^ Using this procedure, vessels form randomly inside the dermis, so they cannot be perfused with a pump from the outside. To solve this drawback, different methods have been developed in order to recreate a perfusable vascular structure inside the dermal compartment.

**FIG. 3.**
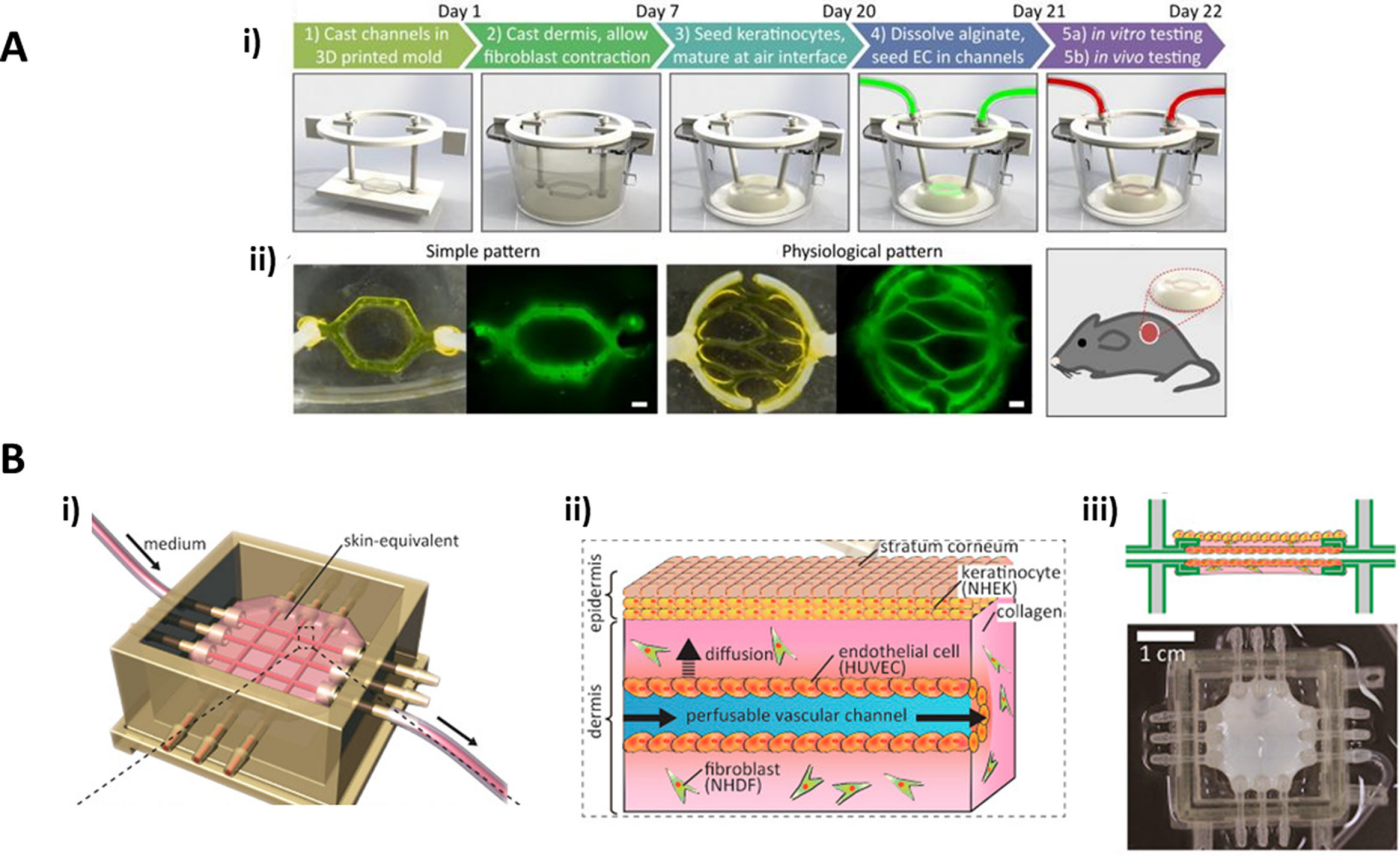
Vascularized skin equivalents. (a) Platform developed by Abaci *et al.*, (i) showing the process for the vascularized skin generation and (ii) showing different vasculature patterns created with the sacrificial alginate networks. Reprinted with permission from Abaci *et al.,* Adv Health Mater. **5**, 14 (2016). Copyright 2021 John Wiley and Sons.^38^ (b) Platform developed by Mori *et al.*, (i) the device structure and layout, (ii) schematic of the obtained skin equivalent, and (iii) lateral layout and top image of the device. Reprinted with permission from Mori *et al.*, Biomaterials **116**, 48–56 (2017). Copyright 2021 Elsevier.^39^

Abaci *et al.*^38^ developed a skin construct built as a classic dermo-epidermal equivalent but introducing different tubular patterns to mimic microvasculature inside the dermal component. They designed a series of 3D printed molds for micropatterning alginate sacrificial channels, which in turn were used for casting a simulated vasculature inside a collagen matrix used as dermal compartment. After epidermal cornification, the alginate was removed from the collagen dermis using sodium citrate, leaving hollow tubules for perfusion [Fig. 3(a)]. Additionally, Abaci introduced either endothelial cells derived from induced pluripotent stem cells (iPSCs) or human umbilical vein endothelial cells (HUVECs) to cover the inner surface of these channels. The addition of these cells showed a decrease in both the permeability and the diffusivity of the microchannels compared to the ones of uncovered vessels and showed more similar values to those of real microvasculature described in previous literature. They also reported an increased neovascularization when the inner-covered constructs were grafted onto immunodeficient mice that was not present using the non-covered ones at 14 days.

Less than a year later, Mori *et al.*^39^ designed a similar skin construct based on a collagen matrix, introducing microvasculature utilizing nylon threads that would be later removed to form the channels. In this case, the platform was made by 3D printing, including anchoring structures for keeping the construct fixed to the device. The nylon threads were introduced through the inlet and outlet ports creating a grid-like structure, and the device was treated with O_2_ plasma for increasing adhesion. Once the collagen hydrogel was formed, the nylon threads were removed and the hollow channels were covered with HUVECs. Contrary to the platform developed by Abaci *et al.*,^38^ the channels are formed and covered with endothelium before skin differentiation and cornification [Fig. 3(b)]. Percutaneous absorption was measured by applying caffeine and isosorbide dinitrate (ISDN) solutions to the upper surface of the skin and measuring the presence of these molecules in both the medium flowing through the microvasculature and the one in the bottom of the culture device. This study was performed with and without VEGF stimulation, showing that this factor affects the vascular permeability of the equivalent. They also studied diffusion from vascular channels and skin permeability, with enhanced results compared with static skin equivalents. Sometime later, Mori *et al.* improved their design by substituting the previous rigid device with a flexible new one, and they introduced a motor to the system. With this new approach, they were able to apply a mechanical force to the tissue, recreating skin stretching and showing that these stimuli enhance the dermal compartment and improve epidermal differentiation and stratification. They also found grooves formation that they relate to early stage wrinkles caused by stretching.^40^

Additionally, other research groups combined the concepts of dynamic vascularized skin models with 3D bioprinting. Kim *et al.*^41^ focused on developing a complex skin construct, including the hypodermal compartment. All the process was carried out combining conventional 3D printing for the fabrication of the device and 3D bioprinting for the generation of the different skin layers. Furthermore, decellularized hypodermal and dermal extracellular matrices (dECMs) were used for casting the corresponding compartments. For vasculature generation, a bioink composed of gelatin, glycerol, and thrombin with endothelial cells embedded was printed with a cylindrical shape; once finished, the construct was incubated at 37 °C, eliminating the gelatin and leaving hollow tubes inside the tissue. Proper tissue formation and maturation are shown in this work, along with good vascular permeability properties that could lead to a promising platform for drug and cosmetic testing and skin diseases modeling.

## IV. ORGAN-ON-A-CHIP

Limitations on traditional 2D cultures and 3D organ models have opened the door to the use of technologies such as microfabrication for biological purposes. This approach has led in the last decade to the development of the so-called organs-on-chips. These are microfluidic devices with micrometer-sized chambers that allow the dynamic culture of cells inside, in order to model or mimic the physiology of a tissue or organ.^42^

The possibility of applying different physical or chemical stimuli to the tissue inside the chip might help with the recreation of its physiology in a more accurate manner than that of a static and traditional 3D culture, having a better control of the cell microenvironment.^43–47^ Additionally, it has been demonstrated that the application of all of these stimuli leads to changes in cell behaviors, showing improved cell differentiation, cell-cell and cell-matrix interactions, and cell morphologies.^48^ These microfluidic devices have shown a huge potential in the study of organ physiologies and the modeling of different diseases^49,50^ as evidenced by the amount of models developed for a great variety of organs: heart,^51^ lung,^52^ intestine,^53^ kidney,^54^ liver,^55^ etc. Nevertheless, most of these organs have been modeled simplifying their 3D structure to single cell monolayers.^47,56–58^

Although these organs-on-chips can be used in different ways depending on the final objectives of the research, the use of porous substrates dividing the microchannels has arisen as a common practice for studying tissue barrier functions and simulating tissue–tissue interfaces.^46,57^ This approach and the micro-scaling of the tissues from 3D traditional cultures to organs-on-chips present two main advantages: first, the transport of substances is more physiologically relevant granting a more realistic evaluation of parameters such as molecules toxicity, or delivery^59^ and second, microfluidics maintain the high throughput capacity of the systems while reducing costs and reagent volumes needed for the experiments.^60^

These two advantages turn organs-on-chips into ideal candidates for drug screening and the discovery and repositioning of pharmacologically relevant molecules.^61,62^ Furthermore, the possibility of interconnecting several of these organ models in the so-called bodies-on-chips allows the study of the systemic responses of several organs to the candidate drug.^63^ The integration of microsensors in the chip for measuring different parameters such as fluid pressures,^64^ cell migration,^65^ or metabolic products^66^ reinforces the utility of these devices in the pharmacology field.

## V. SKIN-ON-A-CHIP

The new advantages offered by organ-on-chip technologies and the necessity of more reliable skin models for drug and cosmetic testing motivated the development of the so-called skin-on-a-chip. These microfluidic devices allow the culture of this tissue under the control of several physical and biochemical parameters as flows, forces, or chemical gradients.^67^ It is not easy to classify all skin-on-a-chip approaches into groups since they differ widely in the main aspects such as the fabrication process and materials or the tissue maintenance. In this Perspective, we have classified the devices according to how the skin is generated in the chip. Two different approaches have been mainly developed to design microfluidic chips for modeling skin: the first one is the direct introduction of a skin fragment coming from a biopsy or a HSE in the chip (transferred skin-on-a-chip) and the second one is focused on the *in situ* generation of the tissue directly on the chip (*in situ* skin-on-a-chip).

### A. Transferred skin-on-a-chip

The most common approaches for skin-on-a-chip models have been those generated by the direct introduction of the tissue inside the device. These transferred tissue fragments have two main origins: a skin biopsy coming from a donor or a HSE that has been generated *in vitro.* Among the models using HSEs for the chips, both laboratory-made equivalents and commercially available ones have been used for skin microfluidic chips. Despite epidermal and dermo-epidermal models can be found in the literature, the ones containing a dermal compartment are more frequently used for transferred skin chips. The use of these well-formed and mature tissue fragments allows more realistic models due to the presence of the different skin layers. Although far from the original definition of organ-on-a-chip, these skin models have permitted the study of different factors affecting the maintenance of the equivalents and their use with clinical and testing purposes (molecules diffusion, multiorgan crosstalk, drugs sensitivity and toxicity, …).

**FIG. 4.**
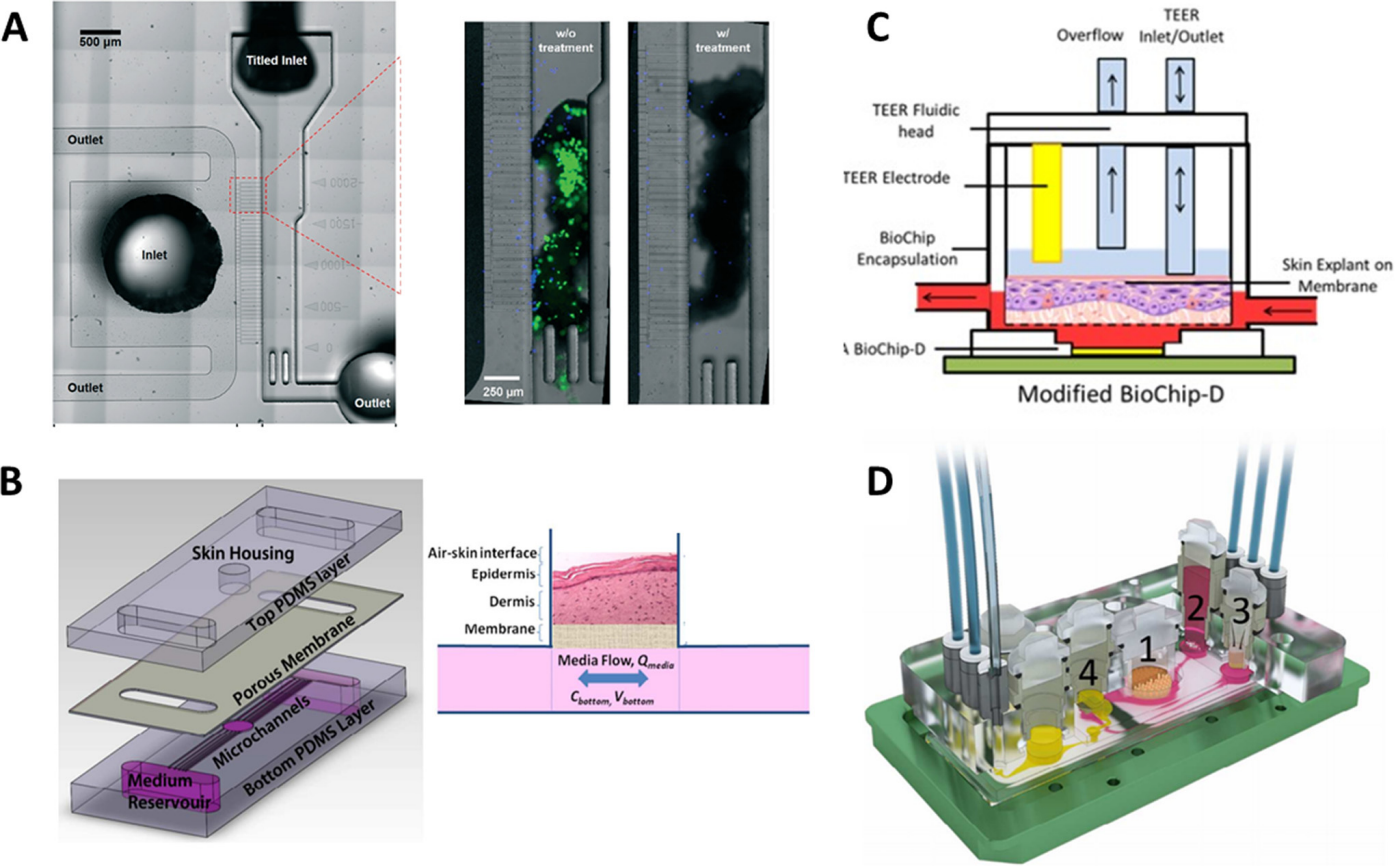
Transferred skin-on-a-chip platforms. (a) Device developed for studying a neutrophil response to skin infection, where the circuit for blood circulation and the channel for the skin biopsy can be appreciated. The magnification shows the presence of bacteria in the skin fragment without and with antibiotic treatment. Republished with permission from Kim *et al.*, Lab Chip **19**, 3094–3103 (2019). Copyright 2021 Royal Society of Chemistry, permission conveyed through Copyright Clearance Center, Inc.^68^ (b) Pumpless chip with transferred skin designed for testing HSEs viability and maintenance. Skin fragment is placed inside the hole in the chip. Republished with permission from Abaci *et al.*, Lab Chip **15**, 3 (2015). Copyright 2021 Royal Society of Chemistry, permission conveyed through Copyright Clearance Center, Inc.^59^ (c) BioChip modified to construct a skin-on-a-chip using EpiDerm™ commercial equivalent. Reprinted with permission from Alexander *et al.*, Genes (Basel) **9**, 2 (2018). Copyright 2018 Authors, licensed under a Creative Commons Attribution (CCBY) License 4.0.^69^ (d) Multiorgan platform including intestine (1), liver (2), skin (3), and kidney (4). Republished with permission from Maschmeyer *et al.,* Lab Chip **15**, 12 (2015). Copyright 2021 Royal Society of Chemistry, permission conveyed through Copyright Clearance Center, Inc.^72^

In order to study neutrophil responses to the presence of bacteria on the skin, Kim *et al.*^68^ designed a single-tissue transferred skin-on-a-chip device with two channels separated by a red blood cell filter. A fragment of a human skin biopsy (previously cultured with bacteria) was introduced in one of the channels and exposed to blood samples loaded in the other one [Fig. 4(a)]. Some research groups use fragments coming from HSEs and not from human biopsies for creating these chips with transferred skin. The chip designed by Abaci *et al.* in 2016^59^ consisted of a well where the HSE fragment was placed for testing its viability and maintenance and a bottom channel for flowing culture medium. The HSE was cultured on top of a porous membrane to allow nutrient diffusion from the channel [Fig. 4(b)]. The group studied the transdermal transport of substances and the capability of using this device for drug testing purposes. In some other cases, already commercially available skin equivalents such as EpiDerm™ were used to create these kinds of chips^69^ [Fig. 4(c)].

Although it has been used for single-tissue models, the transferred skin chip approach is a common practice when developing multi-organ chips. Wagner *et al.*^70^ developed a microfluidic chip for co-culturing skin and liver, introducing directly in the chip a biopsy from human skin, in which they demonstrate a crosstalk between both tissues and liver sensitivity to drug toxicity. In a similar way, Ataç *et al.*^71^ designed a multi-organ chip including hair and skin, in which they modeled skin using a commercial bi-layered equivalent (EpiDermFT™) and subcutaneous tissue coming from human skin biopsies. Using this device, they were able to extend the lifespan of the commercial equivalent, and they demonstrate that the introduction of the subcutaneous tissue improves the viability of the dermo-epidermal construct. Some years later, Maschmeyer *et al.*^72^ created a four-organ system including intestine, liver, skin, and kidney, in which the skin model was also from a human biopsy. They were able to maintain the culture of this system for 28 days, maintaining high cell viability in all tissues [Fig. 4(d)].

### B. *In situ* skin-on-a-chip

This second approach focuses on the generation of the skin model directly on the chip. Two different groups can be also distinguished among the *in situ* skin devices. The first one is similar to those systems discussed before, based on an artificially vascularized dermis: the tissue is generated manually in an open structure inside the device. The main difference relies on how culture medium or any other substances are supplied to the skin construct: while the first ones are perfused through hollow channels passing across the dermal compartment, in these skin-on-chip devices, the circulation of the fluids is carried out through a microfluidic channel below the tissue construct.

**FIG. 5.**
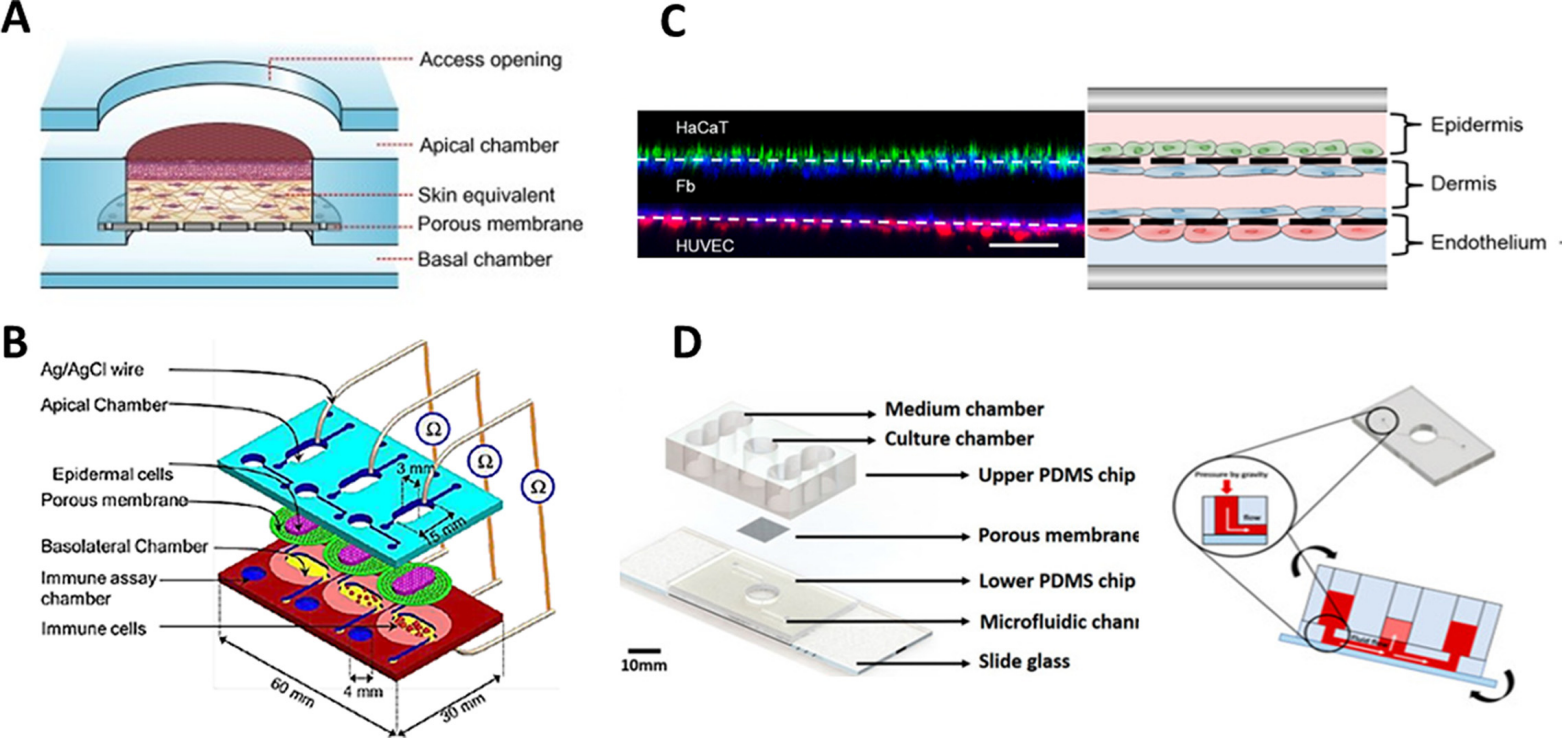
*In situ* skin-on-a-chip platforms. (a) Device composed of two channels and a well-like structure in the middle for casting the dermal compartment. Keratinocytes were inoculated through the upper channel. Reprinted with permission from Sriram *et al.*, Mater Today. **21**, 4 (2018). Copyright 2018 Authors, licensed under a Creative Commons Attribution (CCBY) License 4.0.^80^ (b) Chip designed with two channels and adapted for TEER measurements. Republished with permission from Ramadan *et al.*, Lab Chip **16**, 10 (2016). Copyright 2021 Royal Society of Chemistry, permission conveyed through Copyright Clearance Center, Inc.^82^ (c) Three-layered chip containing keratinocytes, fibroblasts, and HUVECs recreating the three layers of the skin. Reprinted with permission from Wufuer *et al.,* Sci Rep. **6**, 1–12 (2016). Copyright 2016 Authors, licensed under a Creative Commons Attribution (CCBY) License 4.0.^83^ (d) Pumpless microfluidic chip in which skin was directly cast inside the well located in a hole of the middle of the device. Reprinted with permission from Kim *et al.,* Int. J. Mol. Sci. **21**, 11 (2020). Copyright 2020 Authors, licensed under a Creative Commons Attribution (CCBY) License 4.0.^77^

In this direction, Lee *et al.*^73^ developed and optimized a polydimethylsiloxane (PDMS) gravity-driven skin-on-a-chip, in which they constructed the skin directly on a hole inside the device. The dermal compartment, simulated with a collagen gel embedding the fibroblasts, was formed on top of a porous membrane. Then keratinocytes were seeded and differentiated on top of the gel creating a mature skin. Once the device and all the parameters were optimized, Song *et al.*^74^ used the chip for testing how collagens from different sources affected cell differentiation and skin maturation, showing that rat tail collagen gives better results than porcine skin or duck feet collagens. The same group used this device for comparing conventional transwell skin cultures and static and dynamic microfluidic chips.^75^ Although they found good and useful conclusions, the static conditions gave better results than those of the dynamic chip, showing that their model still needed to be optimized. This skin-on-a-chip model was further used for different drug testing. Jeon *et al.*^76^ studied the dermatological side effects of sorafenib, a therapeutic agent used for hepatocellular carcinoma treatments, administering the drug on the skin-on-a-chip model. It was also used by Kim *et al.*^77^ to study the effect of an extract coming from *Curcuma longa* leaves on skin forming and differentiation, finding anti-ageing effects [Fig. 5(d)].

In a similar way, Lim *et al.*^78^ designed a device for both holding the skin equivalent and later applying mechanical stimuli to the tissue. The tissue was generated manually inside a cell chamber over a microfluidic channel, but in this case, an electromagnet was included in the chip structure. This magnet was used to stretch the tissue by applying a magnetic field. Results showed that this mechanical stimulus generated wrinkles on the surface of the equivalent, relating this fact with skin aging, although they described completely contrary results to those obtained by Mori *et al.*^40^ when applying stretching to the skin.

The second *in situ* approach and the ideal aim for skin-on-chips are the modeling of the organ directly inside the device, using the channels not only as a way of delivering nutrients but also as compartments for holding the tissue. As a first approach, keratinocyte culture in microfluidic devices was carried out seeding these cells on collagen covered glass slides and studying how flows affected their growth and viability.^79^ The application of a microflow allowed longer cultures and maintained keratinocytes viability compared to a traditional static culture although the growth rate was reduced. This device cannot be considered as a skin-on-a-chip, but it sets the basis for future skin microfluidic devices.

The use of the channels as compartments for culturing the tissue presents some drawbacks related to the complexity of recreating the 3D structure of the skin. Sriram *et al.*^80^ solved the problem by designing a device consisting of two channels separated by a porous membrane and a well-like structure. The skin was formed in two different steps: the dermal compartment based on fibrin and polyethylene glycol (PEG) was introduced through a hole in the top of the device pipetting the gel; four days after dermis generation and once the device was closed, keratinocytes were inoculated through the upper channel using the inlets of the device. In this approach, the dermal compartment was formed by normal casting while the epidermal one was introduced by means of microfluidics [Fig. 5(a)]. Using this method, they achieved a well-differentiated skin and demonstrated that the microfluidic culture enhances the barrier function of the tissue and favors the synthesis of basement membrane proteins.

This platform solved the problem of recreating the dermo-epidermal structure, although it still depended on generating the dermal construct manually in an open device before the injection of the keratinocytes through the upper channel. The design of models that recreates the skin using only the channels as tissue culture compartments has been studied, but the majority of the devices are based on cellular monolayers. Sasaki *et al.*^81^ developed a photolithography-free microfluidic chip based on PDMS containing several channels allowing parallel experiments. They used this device for cell viability and permeation assays using immortalized keratinocytes monolayers, testing the effect of an allergen for dermatitis (potassium dichromate) on the permeability of the monolayer.

In order to measure the barrier function of a monolayer of keratinocytes, Ramadan and Ting^82^ designed a microfluidic device with two channels separated by a porous membrane [Fig. 5(b)]. They seeded immortalized keratinocytes in the upper channel to form a monolayer on the porous membrane, inoculating through the lower channel a leukemic monocyte lymphoma cell line (U937). Once keratinocytes reached confluence, they applied lipopolysaccharides (LPSs) and nickel sulfate for cell stimulation and posterior cytokine release measurement. Compared with devices only cultured with keratinocytes or immune cells, they found a robust barrier function of the monolayer due to a decrease in monocytes cytokines production.

The first device that more resembled the real architecture of the skin using the channels for containing the tissue was the one developed by Wufuer *et al.*^83^ Although it is mimicked using only cell monolayers, the device has three channels to mimic both the dermis and epidermis and a blood vessel, each of them separated by porous membranes. Immortalized keratinocytes were seeded on top of the upper membrane and HUVECs on the bottom of the lower membrane, simulating the epidermis and the endothelium covering the vessels, respectively. The dermal compartment of the skin was modeled by creating fibroblasts monolayers on the bottom surface of the top membrane and the upper surface of the bottom one using the middle channel [Fig. 5(c)]. The device was utilized for simulating inflammation introducing TFN-α through the middle channel and measuring the production of several cytokines and chemokines, showing that the chip is useful for modeling inflammatory processes. Additionally, the protective effect of dexamethasone (Dex) is studied as a treatment against inflammation, giving the device a role in the drug testing field.

Recently, a work by Risueño *et al.*^84^ presented a skin-on-a-chip device based on adhesive vinyl instead of PDMS that was able to replicate a simplified dermo-epidermal construct inside the microfluidic channels using a fibrin gel as a dermal 3D compartment with an undifferentiated keratinocyte layer on top of it. The fibrin gel was introduced using a parallel flow that allowed generating a dermal compartment with an established and personalized height, leaving enough space in the same channel for keratinocyte seeding, growth, and differentiation. Although it is a very preliminary work since no epidermal differentiation nor tissue characterization is presented, it showed the first skin-on-a-chip generating a 3D structure directly inside the channels of the device.

## VI. CHALLENGES AND FUTURE PERSPECTIVES

Current limitations in traditional skin equivalents' methods have led to the development of new platforms mimicking human physiological conditions and thus enabling the development of more realistic testing models *in vitro*. In addition to new cell types and biological molecules found in a normal skin in order to improve their dermatological performance, new methods have also been developed to emulate the dynamic component present in human beings. Various studies have assessed this issue by developing perfusable human skin equivalents, which mainly consist of introducing a microvasculature in the dermal compartment allowing the injection of drugs or culture medium to study their diffusion and latter effects. Recently, skin tissue engineering has been improving, and other approaches have been developed in order to enhance its performance such as 3D skin bioprinting.^41^ One of its main limitations is that it is still a skin equivalent of large dimensions, which increases the production and maintenance costs and it does not permit to perform high-throughput studies. For this reason, there is an increasing interest in the development of skin-on-a-chip technology to perform real-time monitoring of a large number of specimens in a highly automatized manner reducing the production costs. Traditional lithography technologies have been widely used for the fabrication of PDMS microfluidic devices, although new approaches have been addressed to reduce the costs and the time needed to obtain a chip. Those include the use of new materials such as PMMA or PVC^80,84^ and the application of different micropatterning technologies.^85^

To the best of our knowledge, there are two main different ways of generating skin-on-a-chip: transferred skin-on-a-chip and *in situ* skin-on-a-chip. The first one consists of introducing a skin biopsy or a human skin equivalent in a microfluidic system and subjecting it to a dynamic flow of culture medium through a lower channel. The most meaningful result is a significant improvement in the increase in the skin equivalents or biopsies lifespan for testing purposes. The second one lies on generating the skin model directly inside the microfluidic system. This last approach, which actually should be considered as a proper skin-on-chip, entails many difficulties, still to be addressed. There is a degree of uncertainty around the definition of organ-on-a-chip, however, if we focus on the definition given by Bhatia and Ingber,^42^ who can be considered the father of this technology, only the works developed by Wufuer *et al.*,^83^ Ramadan and Ting,^82^ and Risueño *et al.*^84^ could be purely considered as skin-on-a-chip. In spite of this, the first one reproduced the three layers of the skin as three cell monolayers separated by a porous membrane, while the second only modeled an epidermal monolayer. From a biological point of view, skin is a three-dimensional tissue composed of different layers and various cell types organized in a very determined manner, with a relevant and complex crosstalk between keratinocytes and fibroblasts that has to be considered when designing these models.^5^ Nevertheless, most researchers have simplified the skin model to single monolayers for the difficulties entailed during the cell seeding process. For this reason, the general trend is to manually introduce the cells inside the microfluidic channels using a micropipette instead of using controlled and automated systems such as syringe pumps. The third one is a recently published study that presents a novel methodology where a 3D simplified dermo-epidermal construct is formed inside the microfluidic chip by means of syringe pumps avoiding manual manipulation of the seeding process. Nevertheless, this work is very preliminary since no epidermal differentiation nor tissue characterization is shown.

Another drawback present in the generation of miniaturized skin equivalent in a microfluidic system is the differentiation times needed to develop a complete mature skin structure. In traditional skin culture systems, a fully differentiated and cornified epidermis requires at least three weeks. Sriram *et al.*^80^ reduced epidermal differentiation times to two weeks and faithfully recapitulated the dermo-epidermal junction and enhanced epidermal barrier function. The perfusion of air in the upper compartment was proposed as a possible mechanism to promote epidermal differentiation and cornification instead of just exposing the epidermis to air-liquid interface. This air pumping seems to improve and reduce the time required for that process.

Numerous studies of traditional skin culture systems have also reported a fibroblast-mediated matrix contraction and matrix degradation limiting their lifespan and their reliable application due to the lack of reproducibility. Moreover, researchers have also shown poor mechanical properties, acute shrinkage, and a lack of attachment to the culture insert of the skin constructs. To overcome these problems, they proposed chemical and physical modification of the matrix adding synthetic or natural polymers. Therefore, if the dermal compartment thickness is reduced, there might be technical troubles in developing a fully functional skin inside a chip. Furthermore, working with primary cells is a key point for the correct development of a physiologically relevant model of skin. Protocols to obtain HSE are complex and require specialized workers to do it. Also, the use of specific cell culture media or the culture exposure to an air-liquid interface to induce differentiation and cornification are critical steps to obtain skin equivalents that faithfully resemble a real human skin.

Similarly, there would be some limitations verifying the correct skin differentiation and structure. Traditionally, it has been accomplished, and it still continues to be, by histological sections and immunochemistry. This method implicates the complete removal of the tissue sample from the chip and the end of the experiment. Most of the studies performed in a chip until now have used fluorescent-labeled cells for visual inspection under the microscope. Sriram used a more sophisticated imaging technique like two-photon microscopy complemented with histological images. Wufuer and Ramadan analyzed the cell tight junctions by traditional immunocytochemistry by perfusing the antibodies directly on the chip. Besides, the second one complements the analysis with transepithelial electrical resistance (TEER) measurements demonstrating a better performance when compared to static cultures.

This fact led to the development of biosensors to monitor the state of the skin in real time and also to follow up drug administration and its possible effects. They can be completely integrated into the chip or can be found outside analyzing the fluid coming from the outlet. The latest are protected from fouling or contamination, but they entail an analysis delay. Instead, integrated biosensors bring direct and fast measurements in analytes level changes. Ramadan and Sriram introduced electrodes inside the microfluidic chip, which allowed a real time monitoring of the proliferation and differentiation of the skin equivalent by TEER measurements. In addition, Sriram performed Raman spectroscopy measurements to evaluate the water/keratin content on the skin equivalent that can be related to the *stratum corneum* formation although skin samples must be removed from the chip. Wufuer, in turn, induced an inflammation and edema process and analyzed the skin model response by PCR analysis of cytokine expression levels collected from the outlet fluids. Still, there is a lack of integrated biosensors inside the organ-on-a-chip platforms. The future could lead to electrochemical, optical, or even physical sensors that permit real-time monitoring of the processes that take place during skin equivalent formation or drug administration.

Given the existing technical and biological difficulties found in skin-on-a-chip technology, it is necessary to reason which of the existing techniques would be useful regarding the purpose of the research. In our opinion, traditional 3D skin equivalents and perfusable skin equivalents, despite their limitations (high production costs and the need for specialized personnel), can be useful to study skin diseases, skin irritation or allergies, and drug diffusion; however, they can be structurally and physiologically more complex although their lifespan for testing purposes is very limited in time. Some available skin equivalents are only composed of a cornified epidermis without dermal compartment. This would be enough for testing the skin barrier function and would avoid the aforementioned problems regarding structural stability of the scaffolds but still have several limitations. These factors led to the development of transferred skin-on-chip to extend the time viability of the sample enabling longer experiments. In contrast, skin-on-chip models are suitable for drug testing on a specific cell type to evaluate their response, biocompatibility, or toxicity in a cell monolayer, which is easier to obtain than a 3D equivalent. Additionally, skin-on-chip is the best platform to study cell–cell interactions, to expose cells to mechanical strains, or even to study immune response. For this reason, the development of miniaturized skin tissues on chip provides many advantages, since they are portable, cost-effective, and able to better reproduce the tissue physiological environment and measure drug efficiency rapidly for the skin model. Furthermore, it allows the possibility of high-throughput platforms, where several conditions can be monitored at the same time under controlled parameters. However, the currently used technology and methods have to evolve as it is still not well established how to generate and maintain the skin models and perform the tests inside these microfluidic devices. Nonetheless, this is a powerful and very promising technology and will change the biomedical field of drug discovery and testing.

## Data Availability

Data sharing is not applicable to this article as no new data were created or analyzed in this study.
